# Copper diethyldithiocarbamate as an activator of Nrf2 in cultured vascular endothelial cells

**DOI:** 10.1007/s00775-016-1337-z

**Published:** 2016-01-29

**Authors:** Tomoya Fujie, Masaki Murakami, Eiko Yoshida, Tadashi Tachinami, Yasuhiro Shinkai, Yasuyuki Fujiwara, Chika Yamamoto, Yoshito Kumagai, Hiroshi Naka, Toshiyuki Kaji

**Affiliations:** Department of Environmental Health, Faculty of Pharmaceutical Sciences, Tokyo University of Science, 2641 Yamazaki, Noda, 278-8510 Japan; Graduate School of Science and Research Center for Materials Science, Nagoya University, Chikusa, Nagoya, 464-8602 Japan; Environmental Biology Laboratory, Faculty of Medicine, University of Tsukuba, 1-1-1 Tennodai, Tsukuba, 305-8575 Japan; Department of Environmental Health, School of Pharmacy, Tokyo University of Pharmacy and Life Sciences, 1432-1 Horinouchi, Hachioji, 192-0392 Japan; Department of Environmental Health, Faculty of Pharmaceutical Sciences, Toho University, 2-2-1 Miyama, Funabashi, 274-8510 Japan

**Keywords:** Copper(II) bis(diethyldithiocarbamate), Bio-organometallics, Nrf2, Proteasome, Keap1, Endothelial cell

## Abstract

The interest in organic–inorganic hybrid molecules as molecular probes for biological systems has been growing rapidly. Such hybrid molecules exhibit unique biological activities. Herein, copper(II) bis(diethyldithiocarbamate) (Cu10) was found to activate the transcription factor NF-E2-related factor 2 (Nrf2), which is responsible for regulating antioxidant and phase II xenobiotic enzymes, in vascular endothelial cells. The copper complex rapidly accumulated within cells and induced nuclear translocation of Nrf2, leading to upregulation of the expression of downstream proteins without cytotoxic effects. However, while copper bis(2-hydroxyethyl)dithiocarbamate activated Nrf2, copper ion, diethyldithiocarbamate ligand with or without zinc or iron failed to exhibit this activity. Intracellular accumulation of Cu10 was higher than that of Cu(II) and Cu(I). While the accumulation of copper(II) bis(dimethyldithiocarbamate) was reduced by small interfering RNA (siRNA)-mediated knockdown of the copper transporter CTR1, the knockdown did not affect Cu10 accumulation, indicating that Cu10 rapidly enters vascular endothelial cells via CTR1-independent mechanisms. In addition, copper and iron complexes with other ligands tested could not activate Nrf2, suggesting that the intramolecular interaction between copper and dithiocarbamate ligand is important for the activation of the transcription factor. Cu10 induced the expression of heme oxygenase-1, NAD(P)H quinone oxidoreductase 1, and γ-glutamylcysteine synthetase, downstream proteins of Nrf2. It was suggested that Cu10-induced activation of Nrf2 was due to proteasome inhibition as well as binding to Kelch-like ECH-associated protein 1. Since the effects of Cu10 on vascular endothelial cells are unique and diverse, the copper complex may be a good molecular probe to analyze the functions of the cells.

**Electronic supplementary material:**

The online version of this article (doi:10.1007/s00775-016-1337-z) contains supplementary material, which is available to authorized users.

## Introduction

NF-E2-related factor 2 (Nrf2) is a transcription factor that belongs to Cap‘n’Collar transcription factor family and has a basic leucine zipper domain [1]. Under basal conditions, Nrf2 is bound to Kelch-like ECH-associated protein 1 (Keap1), which is an adapter protein to Cullin3-based E3 ubiquitin ligase, to prevent the proteasomal degradation of Nrf2 in the cytoplasm [2]. Keap1 also functions as a sensor protein against electrophiles and reactive oxygen species. Modification of the reactive thiols of Keap1 by electrophiles results in the dissociation of Nrf2 from Keap1 and its nuclear translocation, allowing it to bind antioxidant response element (ARE) of the genes, thereby forming a heterodimerized complex of Nrf2 with co-activators such as small Maf [3].

Nrf2 mainly regulates the gene expression of antioxidant and phase II xenobiotic metabolizing enzymes such as heme oxygenase-1, NAD(P)H quinone oxidoreductase 1, and γ-glutamylcysteine synthetase, by binding to the ARE of the promoter region of the genes. Induction of heme oxygenase-1 protects cells from oxidative injury by catalyzing heme to biliverdin, carbon monoxide, and iron [4]. NAD(P)H quinone oxidoreductase 1 catalyzes the detoxification of quinones and their derivatives [5]. γ-Glutamylcysteine synthetase is a rate-limiting enzyme in glutathione synthesis and consists of two subunits: the modifier subunit and the catalytic subunit. We postulate that low-molecular-weight molecular probes that activate Nrf2 and regulate cellular functions will be useful in analyzing the involvement of the transcription factor in the regulation of vascular endothelial cell functions.

Organic–inorganic hybrid molecules—organometallic compounds and metal coordination compounds—consist of metals and organic ligands in a common feature. These compounds can exhibit unique biological activities, different from those of organic and inorganic compounds; their activities are attributable to their unique three-dimensional structures and electronic states [6–8]. It is most likely that organic–inorganic hybrid molecules exhibit their activities by modifying the activities of ligand, those of metal, or interaction between ligand and metal. We found that bis(l-cysteinato)zincate(II) serves as a specific zinc donor to the metal response element-binding transcription factor-1, a transcription factor containing six C_2_H_2_ zinc finger domains [6]. We have reported that an organobismuth compound—tris[2-(*N,N*-dimethylaminomethyl)phenyl]bismuthane—exhibits vascular endothelial cell-specific toxicity [7] and the cytotoxicity disappears when the bismuth atom is replaced with an antimony atom [8]. Recently, it was found that an organoantimony compound—tris(pentafluorophenyl)stibane—causes transcriptional induction of metallothionein (submitted).

There are many reports on low-molecular-weight compounds including toxic metal(loid)s that activate Nrf2; for example, sulforaphane, curcumin, *tert*-butylhydroquinone, 1,2-naphthoquinone, methylmercury, and arsenite [9–13]. However, little is known about organic–inorganic hybrid molecules. In the present study, to obtain a good molecular probe for analysis of vascular endothelial cell functions that are regulated by Nrf2, we searched for organic–inorganic hybrid molecules that activate Nrf2 without cytotoxicity in cultured vascular endothelial cells. We found that copper(II) bis(diethyldithiocarbamate) (Cu10) exerts such a biological activity via proteasome inhibition as well as Keap1 modification in the cells.

## Materials and methods

### Materials

Bovine aortic endothelial cells were purchased from Cell Applications (San Diego, CA, USA). The following materials were purchased from the respective vendors: Dulbecco’s modified Eagle’s medium and calcium- and magnesium-free phosphate buffered saline from Nissui Pharmaceutical (Tokyo, Japan); fetal bovine serum from HyClone Laboratories (Waltham, MA, USA); biotin-PEAC_5_-maleimide (BPM) from Dojindo (Kumamoto, Japan); rabbit polyclonal anti-actin antibody (A5060) and copper(II) diacetate (Cu04) from Sigma Aldrich Chemical (St. Louis, MO, USA); horseradish peroxidase-conjugated anti-rabbit IgG antibody (#7074) and anti-biotin, horseradish peroxidase-linked antibody (#7075) from Cell Signaling (Beverly, MA, USA); anti-NAD(P)H quinone oxidoreductase 1 antibody (ab2346) and donkey polyclonal antibody to goat IgG-horseradish peroxidase (ab6885) from Abcam (Tokyo, Japan); rabbit polyclonal anti Nrf2 antibody (H-300), rabbit polyclonal anti CTR1 antibody (FL-190), mouse polyclonal anti Keap1 antibody (H-190), and rabbit polyclonal anti γ-glutamylcysteine synthetase modifier subunit antibody (FL-274) from Santa Cruz Biotechnology (Santa Cruz, CA, USA); rabbit polyclonal anti-heme oxygenase-1 antibody (ADI-SPA-895) and ubiquitin monoclonal antibody (ADI-SPA-203), and MG-132 from Enzo Life Sciences (Farmingdale, NY, USA); nitric oxide, hydrogen peroxide, copper(II) bis(dimethyldithiocarbamate) (Cu17), and sodium diethyldithiocarbamate trihydrate (Na01) from Wako Pure Chemical Industries (Osaka, Japan); 3,5-diaminobenzoic acid, bis(hexafluoroactylacetonato)copper(II) (Cu01), bis(1,3-propanediamine)copper(II) dichloride (Cu07), copper(II) bis(2-hydroxyethyl)dithiocarbamate (Cu09), copper(II) bis(diethyldithiocarbamate) (Cu10), iron(II) phthalocyanine (Fe03), sodium iron(III) ethylenediaminetetraacetate (Fe04), iron(III) tris(diethyldithiocarbamate) (Fe05), nickel(II) bis(diethyldithiocarbamate) (Ni06), zinc(II) bis(diethyldithiocarbamate) (Zn01), zinc(II) bis(dibutyldithiocarbamate), and zinc(II) bis(dibenzyldithiocarbamate) from Tokyo Chemical Industry (Tokyo, Japan); iron(II) diacetate (Fe02) from Acros Organics (Thermo Fisher Scientific, Geel, Belgium); Chemi-Lumi One L and other reagents were from Nacalai Tesque (Kyoto, Japan).

### Synthesis

*N,N*′-Bis(2-methoxycarbonyl-3-oxobutylidene)ethylenediaminatocopper(II) (Cu02), bis(salicylidene)ethylenediaminatocopper(II) (Cu03), and *N*,*N*′-Bis(3,5-di-*tert*-butyl-2-oxidobenzyl)ethylenediaminatocopper(II) (Cu15) were synthesized by employing literature procedures [14–16]. Copper(II) bis(dibutyldithiocarbamate) (Cu18) was synthesized by mixing copper(II) diacetate (3.63 g, 20 mmol) and zinc(II) bis(dibutyldithiocarbamate) (9.48 g, 20 mmol) in a 1:1 molar ratio in a biphasic mixture of dichloromethane (1 L), water (100 mL), and 25 % aqueous ammonia (200 mL) at room temperature for 1 h under aerobic conditions. The organic layer was separated, washed with water, concentrated under reduced pressure, and dried in vacuum to yield the desired product as a black solid (9.32 g, 99 %). Elemental analysis calculated for [C_18_H_36_CuN_2_S_4_]: C, 45.78; H, 7.68; N, 5.93 and found: C, 46.04; H, 7.80; N, 5.65. Copper(II) bis(dibenzyldithiocarbamate) (Cu19) was prepared analogously using zinc(II) bis(dibenzyldithiocarbamate) (92 % yield). Elemental analysis calculated for [C_30_H_28_CuN_2_S_4_]: C, 59.23; H, 4.64; N, 4.60 and found: C, 59.64; H, 4.68; N, 4.24. The elemental analyses were recorded on a Yanaco CHN recorder MT-6 at the Chemical Instrumental Center, Research Center for Materials Science, Nagoya University.

### Western blot analysis

Confluent cultures of vascular endothelial cells in 35-mm culture dishes were incubated at 37 °C for 1, 2, 3, 4, 6, 8, 12, or 24 h with Cu10 or other compounds at 0.1, 0.5, 1, 2, 5, or 10 µM. The cells were washed twice with ice-cold calcium- and magnesium-free phosphate buffered saline; total cellular proteins obtained by lysis in sodium dodecyl sulfate sample buffer (50 mM Tris–HCl buffer solution containing 2 % sodium dodecyl sulfate and 10 % glycerol, pH 6.8) were incubated at 95 °C for 5 min. The protein concentration was determined using a bicinchoninic acid protein assay reagent kit (Thermo Fisher Scientific, Waltham, MA, USA). 2-Mercaptoethanol and bromophenol blue (1.67 % each) were added to the proteins (10 μg). The proteins were separated by SDS–polyacrylamide gel electrophoresis on 10 % polyacrylamide gel and transferred onto a polyvinylidene difluoride membrane at 2 mA/cm^2^ for 1 h. The membrane was blocked with 5 % skim milk in 20 mM Tris–HCl buffer containing 150 mM NaCl and 0.1 % Tween-20, pH 7.5 and incubated with primary antibodies (1:200) at 4 °C overnight. The membrane was washed with 20 mM Tris–HCl buffer solution containing 150 mM NaCl and 0.1 % Tween 20 (pH 7.5), and then incubated with horseradish peroxidase-conjugated secondary antibodies for 1 h at room temperature. Immunoreactive bands were visualized by enhanced chemiluminescence and scanned by LAS3000 (Fujifilm, Tokyo, Japan). Separately, vascular endothelial cells were treated with Cu10 at 0.1, 0.5, 1, 5, or 10 µM for 3 or 6 h and the nuclear fraction was prepared from the cell layer using the NE-PER Nuclear Cytoplasmic Extraction Reagents (Thermofisher Scientific). Nuclear protein concentration was determined by a bicinchoninic acid protein assay reagent kit (Thermo Fisher Scientific). The samples (8 µg protein) were mixed with 50 mM Tris–HCl solution containing 8 % glycerol, 2 % sodium dodecyl sulfate, 2-mercaptoethanol, and 0.005 % bromophenol blue, pH 6.8 and incubated at 95 °C for 3 min. These samples were analyzed by western blotting as described above.

### Intracellular accumulation of metals

Confluent cultures of vascular endothelial cells were incubated in 6-well plates at 37 °C for 3 h in serum-free Dulbecco’s modified Eagle’s medium in the presence of CuSO_4_, sodium diethyldithiocarbamate trihydrate (Na01), zinc(II) bis(diethyldithiocarbamate) (Zn01), iron(III) tris(diethyldithiocarbamate) (Fe05), copper(II) bis(2-hydroxyethyl)dithiocarbamate (Cu09), CuSO_4_ [Cu(II)], CuSO_4_ with 1 mM ascorbate [Cu(I)] [17], or Cu10 (10 µM each). In another experiment, subconfluent cultures of bovine aortic endothelial cells were transfected with control or CTR1 small interfering RNA (siRNA) as described below and incubated at 37 °C for 3 h in the presence of Cu10, Cu17, Cu18, and Cu19 (10 µM each). After incubation, the medium was discarded and the cells were washed twice with ice-cold calcium- and magnesium-free phosphate buffered saline. The cell lysates were prepared by addition of 100 µL 50 mM Tris–HCl containing 2 % SDS and 10 % glycerol (pH 6.8). The cell lysate was incubated at 95 °C for 3 min and a portion was treated with nitric acid-H_2_O_2_ at 130 °C for 1 day to degrade proteins and dissolved with 2 mL of 0.1 M nitric acid; the diluted samples were used for determination of zinc, copper, and iron content by inductively coupled plasma mass spectrometry (ERAN DRC II, PerkinElmer, MA, USA). Another portion of the cell lysate was analyzed for DNA content by fluorometric method [18] to normalize the content of the metals per µg DNA.

### Transfection

Vascular endothelial cells were cultured and siRNAs (Bioneer, Daejeon, Korea) for the copper transporter CTR1 were transfected using RNAiMAX reagent (Invitrogen, Crlsbad, CA, USA), as described previously [19]. Briefly, the cells were cultured in Dulbecco’s modified Eagle’s medium supplemented with 10 % fetal bovine serum in 35-mm dishes until 70–80 % confluence. Separately, siRNA duplex (35 pmol/mL) and transfection reagent (2 μL/mL) were mixed with Opti-MEM (Thermofisher Scientific) and incubated for 20 min at room temperature. The mixture was added to the culture medium, and the cells were incubated at 37 °C for 24 h and then treated with or without Cu10, Cu17, Cu18, or Cu19 (10 µM each) for 3 h. The sequences of the sense and antisense strands of siRNA were as follows: bovine CTR1 siRNA, 5-AUAAGGAUGGUUCCAUUUGdTdT-3 (sense) and 5-CAAAUGGAACCAUCCUUAU-3 (antisense). A nonspecific sequence was used as the siRNA negative control (Qiagen, Valencia, CA, USA).

### Keap1 and biotin-PEAC_5_-maleimide-labeling assay in vitro

The mouse recombinant Keap1 construct was prepared as described previously [10, 20]. The recombinant Keap1 protein was expressed as a C-terminal His-tagged fusion protein in BL21(DE3)pLysS *E. coli* cells and purified using a ProBond nickel-resin. The BPM-labeling assay was performed according to the method described by Toyama et al. [20]. Briefly, mouse recombinant Keap1 protein (2 µg) was incubated with Cu10 (1, 10, or 100 µM) at 37 °C for 30 min in 100 mM Tris–HCl buffer solution (pH 7.5). After incubation, 25 µM biotin-PEAC_5_-maleimide was added to the samples and the samples were incubated at 37 °C for 30 min. The samples were electrophoresed on 10 % sodium dodecyl sulfate–polyacrylamide gel in 50 mM Tris–HCl containing 2 % SDS, 8 % glycerol, and 0.005 % bromophenol blue (pH 6.8) without 2-mercaptoethanol and were incubated at 37 °C for 30 min. They were then subjected to immunoblotting as described above.

### Statistical analysis

The data were analyzed for statistical significance by Student’s *t* test when possible. *P* values less than 0.01 were considered statistically significant.

## Results

### Cu10 activates Nrf2 in vascular endothelial cells

**Fig. 1:**
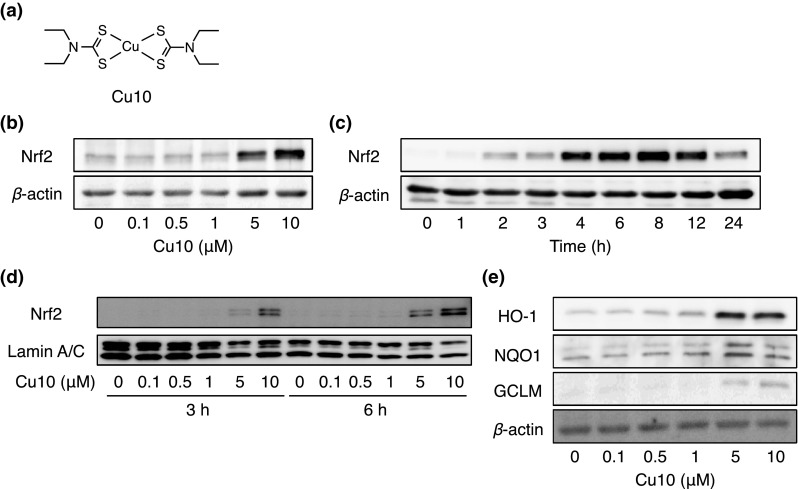
Activation of Nrf2 by Cu10 in vascular endothelial cells. **a** The structure of Cu10. **b** The expression of Nrf2. Confluent cultures of bovine aortic endothelial cells were incubated at 37 °C for 3 h in the presence or absence of Cu10 (0.1, 0.5, 1, 5, or 10 µM). **c** Time course of the effect of Cu10 on the expression of Nrf2. Confluent cultures of bovine aortic endothelial cells were incubated at 37 °C for 1, 2, 3, 4, 6, 8, 12, and 24 h in the presence or absence of Cu10 (10 µM). **d** The expression of Nrf2 in the nuclei. Confluent cultures of bovine aortic endothelial cells were incubated at 37 °C for 3 and 6 h in the presence or absence of Cu10 (0.1, 0.5, 1, 5, or 10 µM). **e** The expression of downstream proteins of Nrf2. Confluent cultures of bovine aortic endothelial cells were incubated at 37 °C for 24 h in the presence or absence of Cu10 (0.1, 0.5, 1, 5, or 10 µM). *HO-1* heme oxygenase-1, *NQO1* (*upper bands*) NAD(P)H quinone oxidoreductase 1, *GCLM* γ-glutamylcysteine synthetase modifier subunit

Figure 1 shows the activation of Nrf2 by Cu10 (the structure is shown in Fig. 1a) in vascular endothelial cells. Cu10 at 10 µM or less increased the expression of Nrf2 in a concentration-dependent manner (Fig. 1b). Cu10 at 10 µM increased the expression of Nrf2 after 2 h or longer in a time-dependent manner; the highest expression was observed at 8 h and gradually reduced thereafter (Fig. 1c). Nrf2 was detected in the nuclear fraction after 3 and 6 h in vascular endothelial cells treated with Cu10 at 5 and 10 µM (Fig. 1d). After a 24 h treatment with Cu10 at 0.1 µM or higher, the expression of downstream proteins of Nrf2—heme oxygenase-1, NAD(P)H quinone oxidoreductase 1, and γ-glutamylcysteine synthetase modifier subunit—significantly increased in a concentration-dependent manner (Fig. 1e).

### Role of copper in the Cu10 molecule in Nrf2 activation

**Fig. 2:**
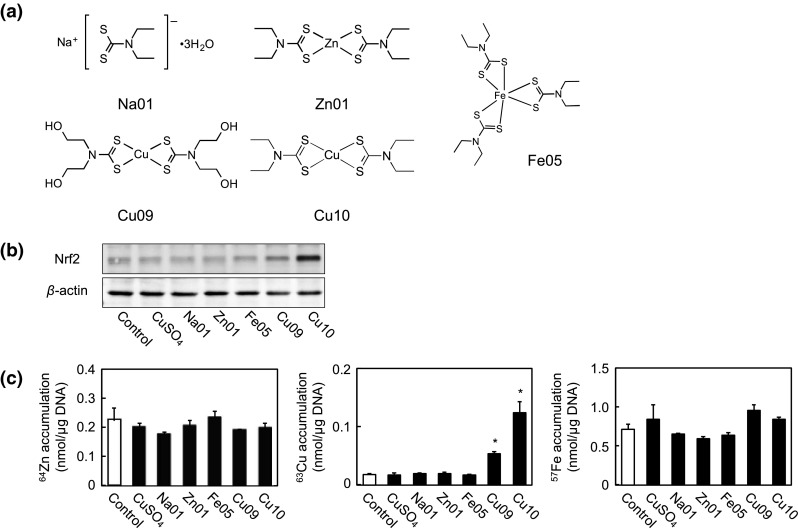
Role of copper in the Cu10 molecule in Nrf2 activation in vascular endothelial cells. **a** The structures of Na01, Zn01, Fe05, Cu09, and Cu10. **b** The expression of Nrf2. Confluent cultures of bovine aortic endothelial cells were incubated at 37 °C for 3 h in the presence or absence of CuSO_4_, Na01, Zn01, Fe05, Cu09, and Cu10 (10 µM each). **c** Intracellular accumulation of CuSO_4_, Na01, Zn01, Fe05, Cu09, and Cu10. Confluent cultures of bovine aortic endothelial cells were incubated at 37 °C for 3 h in the presence or absence of CuSO_4_, Na01, Zn01, Fe05, Cu09, and Cu10 (10 µM each). *White*, *gray*, and *black bars* indicate the content of zinc, iron, and copper, respectively. Values are mean ± SE of three samples. *Significantly different from the control, *P* < 0.01

In order to examine whether copper in the Cu10 molecule is critical to the activation of endothelial Nrf2, the effects of zinc and iron complexes with the same ligand of Cu10 on Nrf2 activation were investigated. In this experiment, copper sulfate, Na01 as the ligand of Cu10, and Cu09 were investigated. The structures of the tested metal complexes and Na01 are shown in Fig. 2a. As shown in Fig. 2b, Cu10 and Cu09 increased the expression of Nrf2; however, copper sulfate, Na01, Zn01, and Fe05 failed to exhibit such an activity (Fig. 2b), indicating that copper is required for diethyldithiocarbamate complexes to activate Nrf2 in vascular endothelial cells. Copper sulfate, Zn01, and Fe05 did not accumulate within the cells after a 3 h treatment, whereas significant accumulation of Cu10 and Cu09 was observed (Fig. 2c), suggesting that Nrf2-activating activity of the copper complexes may depend on their high intracellular accumulation.

**Fig. 3:**
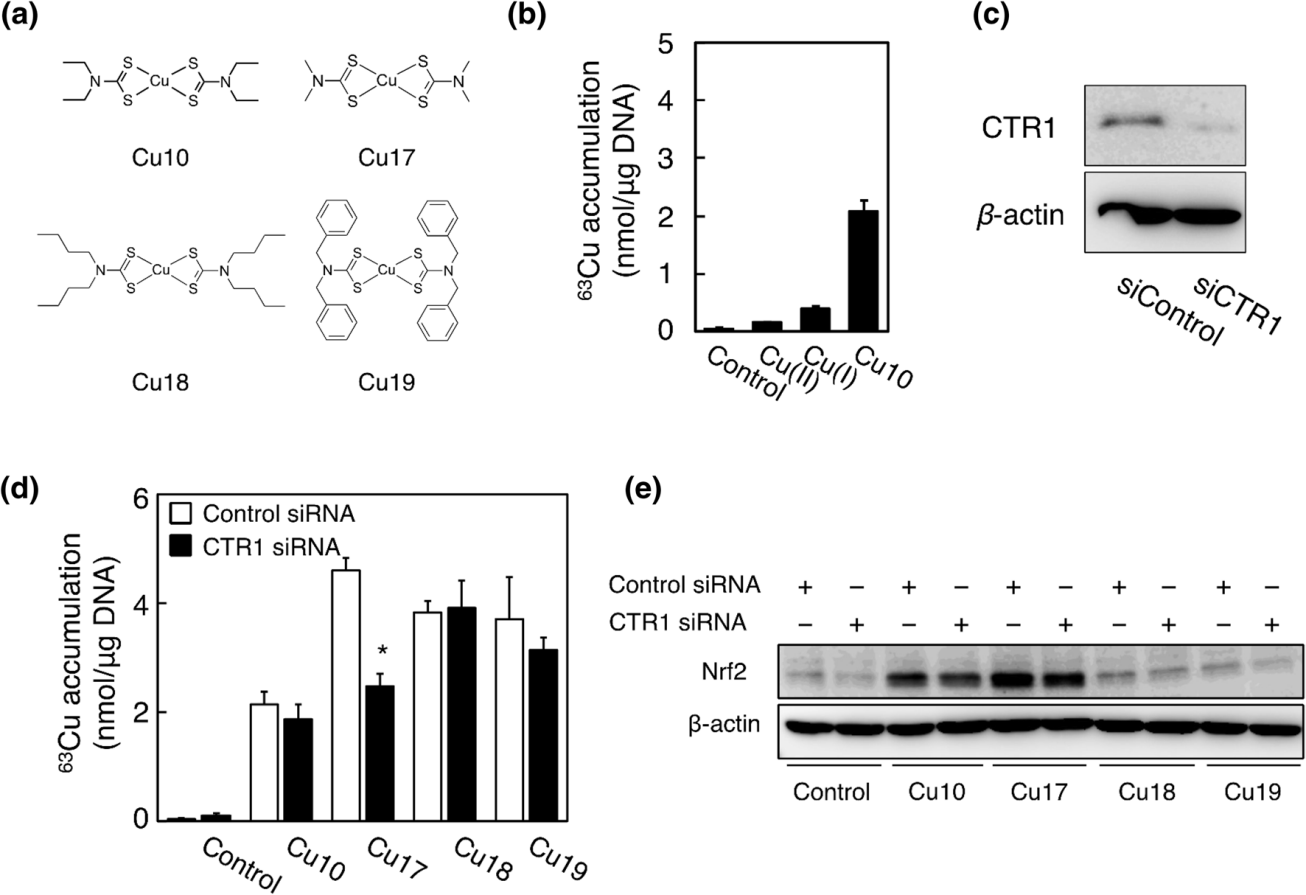
Intracellular accumulation of Cu(II), Cu(I), and Cu10 in vascular endothelial cells, and involvement of CTR1 in the accumulation of copper complexes. **a** The structures of Cu10, Cu17, Cu18, and Cu19. **b** Intracellular accumulation of Cu(II), Cu(I), and Cu10. Confluent cultures of bovine aortic endothelial cells were incubated at 37 °C for 3 h in the presence or absence of CuSO_4_ [Cu(II)], CuSO_4_ with 1 mM ascorbate [Cu(I)], and Cu10 (10 µM each). Values are expressed as mean ± SE for the four samples. **c** CTR1 protein expression after siRNA-mediated knockdown of CTR1. Subconfluent cultures of bovine aortic endothelial cells were transfected with control or CTR1 siRNA and incubated at 37 °C in the presence or absence of Cu10, Cu17, Cu18, and Cu19 (10 µM each) for 3 h. **d** Intracellular accumulation of Cu10, Cu17, Cu18, and Cu19. Subconfluent cultures of bovine aortic endothelial cells were transfected with control or CTR1 siRNA and incubated at 37 °C in the presence or absence of Cu10, Cu17, Cu18, and Cu19 (10 µM each). Values are expressed as mean ± SE for the four samples. *Significantly different from the control, *P* < 0.01. **e** Nrf2 expression. Subconfluent cultures of bovine aortic endothelial cells were transfected with control or CTR1 siRNA and incubated at 37 °C in the presence or absence of Cu10, Cu17, Cu18, and Cu19 (10 µM each)

Since Cu10 and Cu09 highly accumulated within vascular endothelial cells and enhanced Nrf2 expression, Cu(II) may have been reduced to Cu(I) by thiol groups in the thiocarbamate ligands and Cu(I) ion released from the Cu10 molecule efficiently entered the cells through the copper transporter CTR1 that mediated Cu(I) uptake [17]. To examine this hypothesis, we compared the intracellular accumulation of Cu(II), Cu(I), and Cu10 in vascular endothelial cells and determined the involvement of CTR1 in the uptake of copper complexes with thiol groups. The structures of the tested copper complexes are shown in Fig. 3a. The intracellular accumulation of Cu10 was high in vascular endothelial cells compared with Cu(II) and Cu(I) (Fig. 3b). The accumulation of copper(II) bis(dimethyldithiocarbamate) (Cu17) was significantly reduced by siRNA-mediated knockdown of CTR1; however, Cu10, copper(II) bis(dibutyldithiocarbamate) (Cu18), and copper(II) bis(dibenzyldithiocarbamate) (Cu19) accumulation was not affected by the knockdown (Fig. 3c, d). Cu17 as well as Cu10 activated Nrf2, regardless of whether CTR1 was knocked down or not (Fig. 3e). However, Cu18 and Cu19 highly accumulated in the cells (Fig. 3d), regardless of CTR1 expression, but failed to activate Nrf2 in vascular endothelial cells.

### Role of the ligand in the Cu10 molecule in Nrf2 activation

**Fig. 4:**
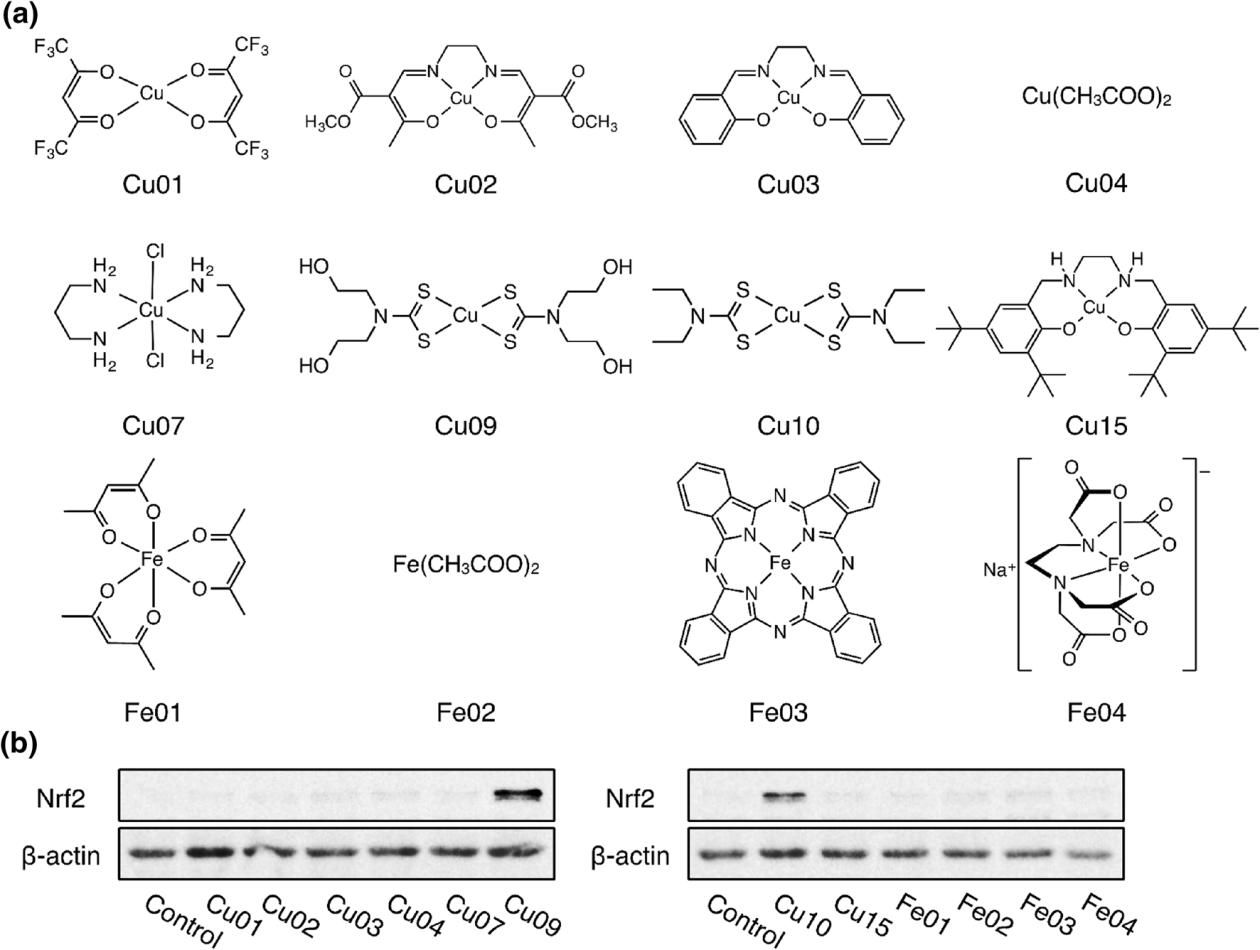
Role of the ligand in Cu10 molecule in Nrf2 activation in vascular endothelial cells. **a** The structures of Cu01, Cu02, Cu03, Cu04, Cu07, Cu09, Cu10, Cu15, Fe01, Fe02, Fe03, and Fe04. **b** The expression of Nrf2. Confluent cultures of bovine aortic endothelial cells were incubated at 37 °C for 3 h in the presence or absence of Cu01, Cu02, Cu03, Cu04, Cu07, Cu09, Cu10, Cu15, Fe01, Fe02, Fe03, and Fe04 (10 µM each)

It is possible that copper complexes in general activate endothelial Nrf2. To examine this possibility, we evaluated copper complexes with various ligands. In this experiment, compounds containing iron were also examined. The compounds used in this experiment are shown in Fig. 4a. Among the tested copper complexes, only Cu10 and Cu09 increased the expression of Nrf2 (Fig. 4b), suggesting that the ligand as well as the copper ion is important for the activation of Nrf2 by Cu10. Other compounds could not activate the transcription factor. The expression of Nrf2 in vascular endothelial cells treated with Cu01, Cu02, Cu03, Cu04, Cu07, Cu09, Cu15, Fe01, Fe02, Fe03, Fe04, Fe05, CuSO_4_, and Na01 is shown in S1.

### Characterization of Nrf2 activation by Cu10

**Fig. 5:**
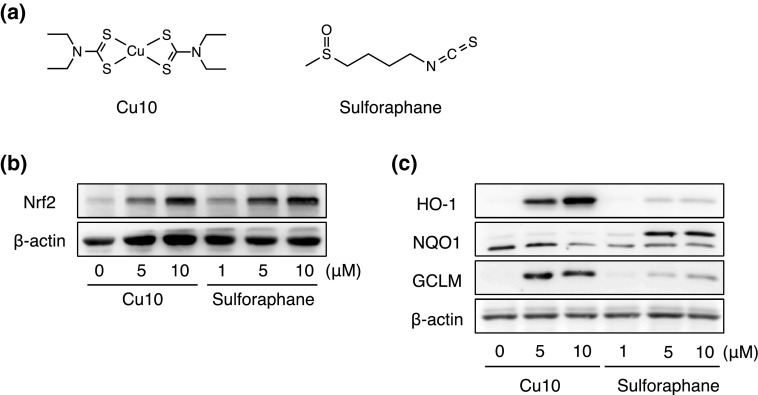
Characterization of Nrf2 activation by Cu10 compared with sulforaphane. **a** The structures of Cu10 and sulforaphane. **b** The expression of Nrf2. Confluent cultures of bovine aortic endothelial cells were incubated at 37 °C for 3 h in the presence or absence of Cu10 (5 or 10 µM) or sulforaphane (1, 5, or 10 µM). **c** The expression of downstream proteins of Nrf2. Confluent cultures of bovine aortic endothelial cells were incubated at 37 °C for 24 h in the presence or absence of Cu10 (5 or 10 µM) or sulforaphane (1, 5, or 10 µM). *HO-1* heme oxygenase-1, *NQO1* NAD(P)H quinone oxidoreductase 1, *GCLM* γ-glutamylcysteine synthetase modifier subunit

The activation of Nrf2 by Cu10 was compared with that by sulforaphane (the structure is shown in Fig. 5a), an isothiocyanate that modifies Keap1 and activates Nrf2 [21]. It was shown that the Nrf2-activating activity of Cu10 and sulforaphane is almost comparable (Fig. 5b); however, the expression of Nrf2 downstream proteins induced by Cu10 and sulforaphane differed. Specifically, Cu10 markedly increased the expression of heme oxygenase-1 and γ-glutamylcysteine synthetase modifier subunit, whereas the expression of NAD(P)H quinone oxidoreductase 1 was markedly upregulated by sulforaphane (Fig. 5c), suggesting that the mechanisms underlying Nrf2 activation by Cu10 may be different from that of sulforaphane.

### Activation mechanisms of Nrf2 by Cu10

**Fig. 6:**
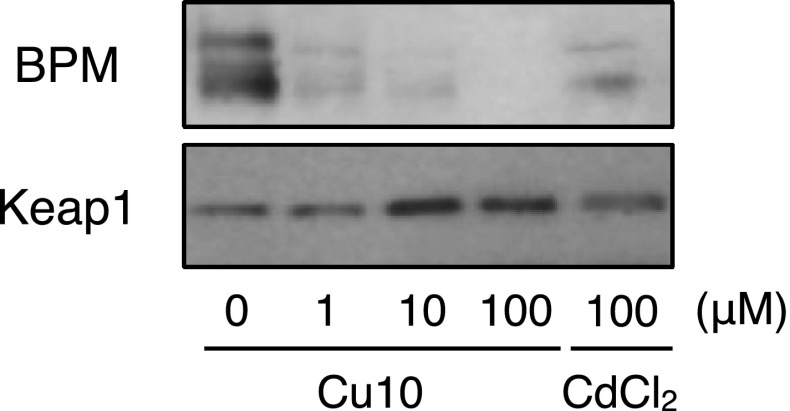
Binding of Cu10 to Keap1. Recombinant mouse Keap1 protein (2 µg) was incubated with Cu10 (1, 10, or 100 µM) at 37 °C for 30 min in 100 mM Tris–HCl (pH 7.5) and then further incubated at 37 °C for 30 min after addition of 25 µM biotin-PEAC_5_-maleimide. The samples were subjected to western blotting, which was performed using anti-biotin antibody (BPM) and anti-Keap1 antibody (Keap1). Cadmium chloride (CdCl_2_) was used as the positive control

**Fig. 7:**
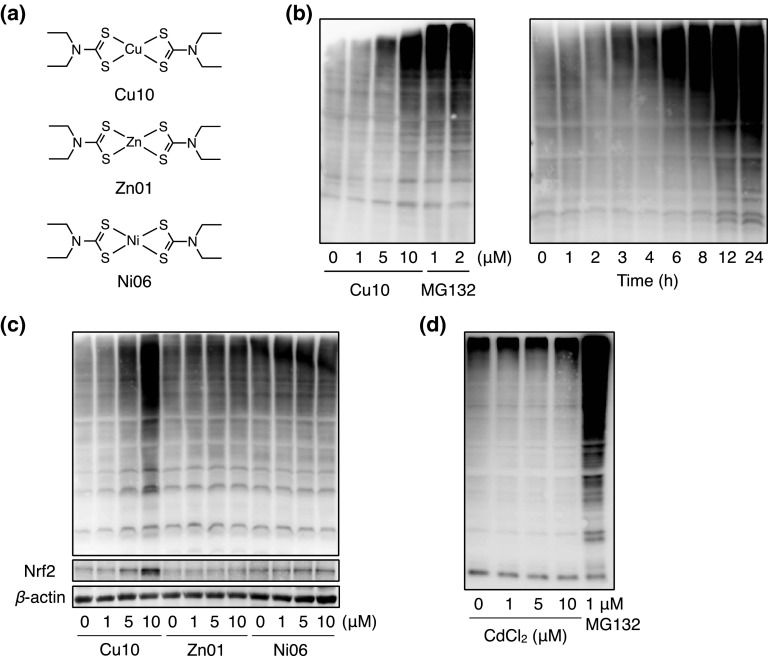
Proteasome inhibition by Cu10, Zn01, Ni06, and CdCl_2_ in vascular endothelial cells. **a** The structures of Cu10, Zn01, and Ni06. **b** Proteasome inhibitory activity. Confluent cultures of bovine aortic endothelial cells were incubated at 37 °C for 8 h in the presence or absence of Cu10, Zn01, Ni06, or cadmium chloride (CdCl_2_) (1, 5, 10 µM each). MG132 was used as positive control. The total cell lysates were subjected to western blotting, which was performed using an anti-ubiquitin antibody

To examine whether Cu10 can bind Keap1, the BPM-labeling assay was performed. Incubation of recombinant mouse Keap1 with Cu10 decreased the signal of biotinylated Keap1 protein (Fig. 6), indicating that Cu10 was bound to Keap1. As shown in the previous study [20], cadmium bound to Keap1. Since Keap1 binds Nrf2 and protects it from proteasomal degradation, inhibition of proteasome can be a mechanism through which Nrf2 is activated. To examine this possibility, the proteasome inhibitory activity of Cu10 was investigated. In this experiment, Zn01 and nickel(II) bis(diethyldithiocarbamate) (Ni06) were also evaluated (the structures are shown in Fig. 7a). It was shown that Cu10 increased the ubiquitinated proteins in a concentration- and time-dependent manner (Fig. 7b), indicating that the copper complex inhibits proteasome; MG132, a typical proteasome inhibitor also increased the level of ubiquitinated proteins. Zn01 and Ni06 failed to increase both Nrf2 expression and level of ubiquitinated proteins (Fig. 7c). Since cadmium, a toxic heavy metal in vascular endothelial cells [22], did not display proteasome inhibitory activity, it is suggested that the proteasome inhibition by Cu10 was not a nonspecific effect of the copper complex (Fig. 7d).

## Discussion

Dithiocarbamates are metal-chelating compounds that affect the activities of various metal-binding proteins such as nuclear factor-kappa B and superoxide dismutase-1 [23, 24]. The present study revealed the previously unrecognized biological activities of metal diethyldithiocarbamate coordination compounds. The following results were obtained: (1) copper(II) bis(diethyldithiocarbamate), Cu10, activates Nrf2 in vascular endothelial cells without cytotoxicity, (2) the combination of copper with the diethyldithiocarbamate ligand is essential for the effect of Cu10, (3) Cu10 rapidly enters the cells and induces nuclear translocation of Nrf2, resulting in the induction of downstream proteins such as heme oxygenase-1, NAD(P)H quinone oxidoreductase 1, and γ-glutamylcysteine synthetase modifier subunit, (4) high concentrations of Cu10 accumulated within the cells compared with Cu(II) and Cu(I), regardless of CTR1 expression, and Nrf2 activation of copper complexes with thiol groups depended on the structures and not on intracellular accumulation; (5) both proteasome inhibition and binding to Keap1 are mechanisms through which Cu10 activates Nrf2. In general, the relationship between the biological activity and the structure of a certain organic–inorganic hybrid molecule could follow any of the following three patterns. First, the ligand has biological activity and intramolecular metal intensifies the activity. Second, the metal has biological activity and the ligand intensifies the activity. Third, the biological activity of either the ligand or the metal is only slight but the hybrid molecule exhibits activity owing to their discrete molecular structures that originate from metal–ligand coordination (i.e., intramolecular interaction). Since neither copper sulfate nor Na01 could activate Nrf2, we postulate that the activation of Nrf2 by Cu10 is induced by the intramolecular interaction between copper atom and the diethyldithiocarbamate ligand. It is also suggested that this interaction is required for rapid accumulation of Cu10 within vascular endothelial cells.

The mechanisms of high intracellular accumulation of Cu10 are critical for understanding the mechanisms by which Cu10 activates Nrf2 in vascular endothelial cells. Copper complexes with thiol groups, such as Cu10 and Cu09, markedly accumulate within the cells and activate Nrf2, whereas copper complexes without thiol groups failed to activate Nrf2, suggesting that Cu(II) may be reduced to Cu(I) by thiol groups in thiocarbamate ligands, and the Cu(I) ion released from the Cu10 molecule efficiently entered the cells via the copper transporter CTR1 [17]. In other words, Cu10 served as a donor of Cu(I) to CTR1, and consequently, the copper ion, but not the Cu10 molecule, activated Nrf2 through modification of Keap1. However, intracellular accumulation of Cu10 was much higher than that of Cu(II) and Cu(I) and was not affected by siRNA-mediated knockdown of CTR1. In contrast, the accumulation of Cu17 was significantly reduced by the knockdown, suggesting that CTR1 is at least partly involved in the uptake of Cu17. Therefore, the possibility that CTR1 partly mediates the uptake of copper complexes as well as cisplatin [25], cannot be excluded, and it is suggested that CTR1 expression is not the major mechanism of Cu10 uptake. In addition, Cu18 and Cu19 were also highly accumulated within the cells, but failed to activate Nrf2, thereby suggesting that activation of Nrf2 by copper complexes depends on the ligand structure rather than the copper ion released from the molecule. Thus, although the details are yet to be elucidated, an assumption can be made that Cu10 was transported as a molecule, and modified Keap1 and activated Nrf2 in vascular endothelial cells.

Ubiquitin–proteasome system is responsible for the degradation of numerous proteins, including Nrf2. The 26S proteasome consists of two complexes—the 20S proteolytic core and the 19S regulatory complex [26]. Previously, it was reported that Cu10 inhibits proteasomal function by inhibiting both 20S chymotrypsin-like activity and 19S complex in human breast cancer MBA-MD-231 cells [27]. In this report, Zn01 but not Ni06 showed proteasome inhibitory activity, suggesting that the type of metal complexes that have similar biological activities may depend on cell types. While the reason underlying this cell type dependency is unclear, it is certain that Cu10 exhibits proteasome inhibitory activity and inhibits the degradation of Nrf2 in vascular endothelial cells. Proteasome inhibition would be one of the major mechanisms by which Cu10 activates endothelial Nrf2. Conversely, it was shown that Cu10 binds Keap1 and this led to the release of Nrf2 from Keap1 followed by nuclear translocation of Nrf2. There are several reactive cysteine residues in the Keap1 molecule and the residues that are involved in Nrf2 activation are Cys151 in the BTB domain and Cys273/Cys288 in the IVR domain [28, 29]. The modified cysteine residues that are employed for Nrf2 activation depend on the compounds that activate Nrf2 [30, 31]. For example, zinc ion binds to both Cys273 and Cys288 and activates Nrf2 [32]. It is suggested that Cu10 binds to at least one of the three reactive cysteine residues of the Keap1 molecule. While it is unclear as to which cysteine residue(s) are modified by Cu10, it is postulated that modification of Keap1 is one of the major mechanisms by which Cu10 activates endothelial Nrf2. It was shown that, among the downstream proteins of Nrf2, heme oxygenase-1 and γ-glutamylcysteine synthetase modifier subunit were markedly induced by Cu10, whereas sulforaphane, which is bound to Keap1 and activates Nrf2, strongly induced NAD(P)H quinone oxidoreductase 1. This difference in the induction of downstream protein between Cu10 and sulforaphane may be attributable to the difference of Nrf2 activation mechanisms. However, this is yet to be elucidated.

A compound that has a specific target biomolecule is an excellent tool to analyze the role of the biomolecule in the regulation of some biological systems. However, a compound that has multiple targets is also useful to analyze the relationship among the targets. Cu10 appears to belong to the latter case. In fact, we have analyzed vascular endothelial cell functions using Cu10. Metallothionein is a low-molecular-weight, cysteine-rich, metal-containing, inducible protein, which protects cells from heavy metals and oxidative stress [33]. Since cadmium and zinc induce metallothionein, they have been used as tools to analyze mechanisms underlying metallothionein induction. However, the metals cannot be good tools because vascular endothelial cells are sensitive to cadmium [34] and zinc does not induce metallothionein in the cells [35, 36]. Recently, we found that this copper complex induces metallothionein in vascular endothelial cells; activation of Nrf2 and consequent activation of ARE in the promoter region of metallothionein genes contribute to the induction of specific metallothionein isoform [19]. Further studies on Cu10 as a tool to analyze vascular endothelial cell functions are ongoing.

## Electronic supplementary material

Supplementary material 1 (PDF 190 kb)

## Abbreviations

ARE: Antioxidant response element
BPM: Biotin-PEAC_5_-maleimide
Cu01: Bis(hexafluoroactylacetonato)copper(II)
Cu02: *N,N′*-Bis(2-methoxycarbonyl-3-oxobutylidene)ethylenediaminatocopper(II)
Cu03: Bis(salicylidene)ethylenediaminatocopper(II)
Cu04: Copper(II) diacetate
Cu07: Bis(1,3-propanediamine)copper(II) dichloride
Cu09: Copper(II) bis(2-hydroxyethyl)dithiocarbamate
Cu10: Copper(II) bis(diethyldithiocarbamate)
Cu15: *N*,*N′*-Bis(3,5-di-*tert*-butyl-2-oxidobenzyl)ethylenediaminatocopper(II)
Cu17: Copper(II) bis(dimethyldithiocarbamate)
Cu18: Copper(II) bis(dibutyldithiocarbamate)
Cu19: Copper(II) bis(dibenzyldithiocarbamate)
Fe01: Tris(acetylacetonato)iron(III)
Fe02: Iron(II) diacetate
Fe03: Iron(II) phthalocyanine
Fe04: Sodium iron(III) ethylenediaminetetraacetate
Fe05: Iron(III) tris(diethyldithiocarbamate)
Keap1: Kelch-like ECH-associated protein 1
MG132: Z-Leu-Leu-Leu-CHO
Na01: Sodium diethyldithiocarbamate trihydrate
Ni06: Nickel(II) bis(diethyldithiocarbamate)
Nrf2: NF-E2-related factor 2
Zn01: Zinc(II) bis(diethyldithiocarbamate)
