# Supplementary figures and images for: Copper diethyldithiocarbamate as an activator of Nrf2 in cultured vascular endothelial cells
Source: J Biol Inorg Chem. 2016 Jan 29;21:263–73. doi: 10.1007/s00775-016-1337-z (PMC4801994; doi:10.1007/s00775-016-1337-z)

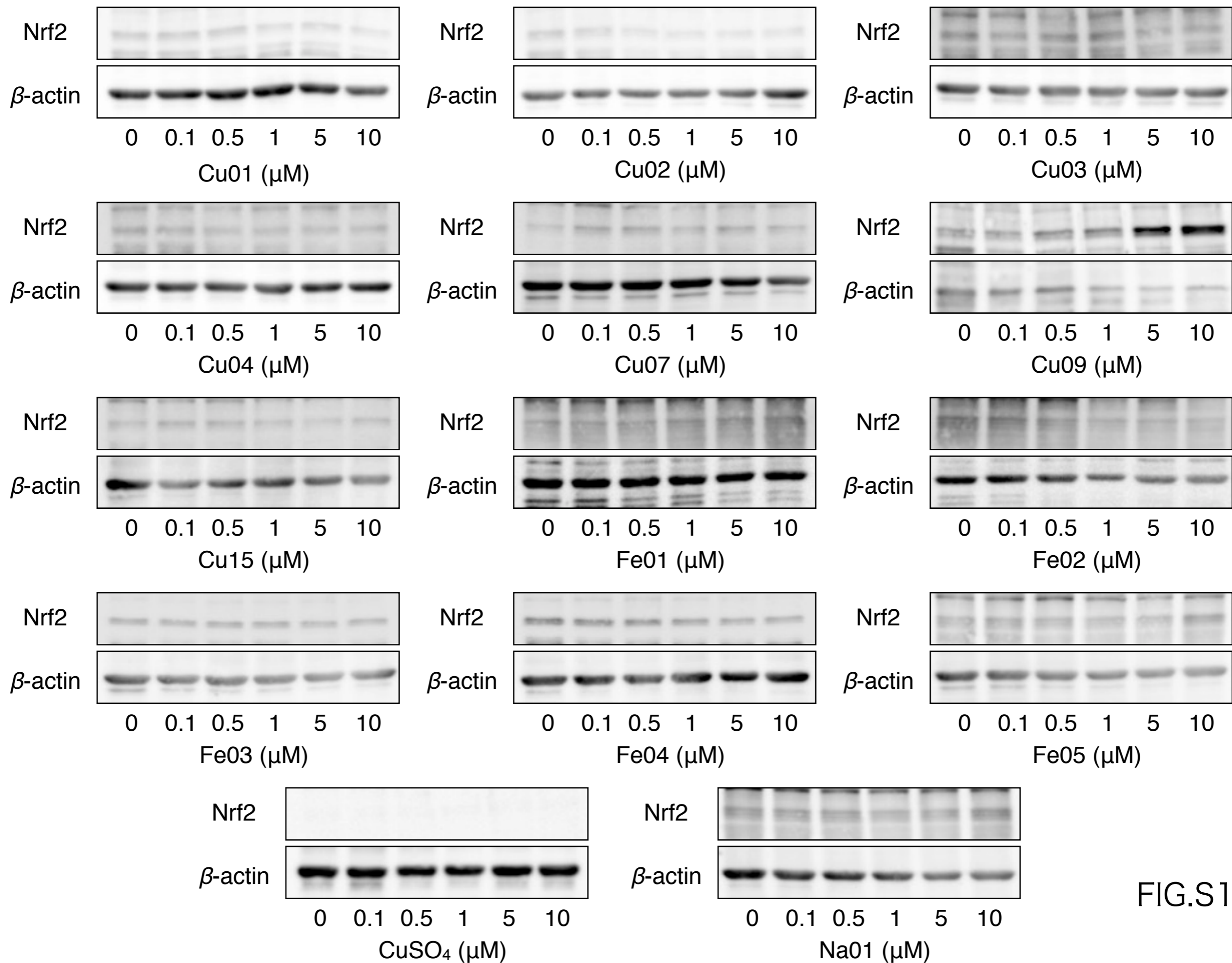

FIG.S1

Supplement: Supplementary file 1 — Supplementary material 1 (PDF 190 kb) [file 775_2016_1337_MOESM1_ESM.pdf]
